# Kaempferol Alleviates Murine Experimental Colitis by Restoring Gut Microbiota and Inhibiting the LPS-TLR4-NF-κB Axis

**DOI:** 10.3389/fimmu.2021.679897

**Published:** 2021-07-22

**Authors:** Yifan Qu, Xinyi Li, Fengying Xu, Shimin Zhao, Xuemei Wu, Yuzhen Wang, Jiming Xie

**Affiliations:** ^1^ Inner Mongolia Clinical College, Inner Mongolia Medical University, Hohhot, China; ^2^ Clinical Laboratory, Inner Mongolia People’s Hospital, Hohhot, China; ^3^ College of Life Science, Inner Mongolia Agricultural University, Hohhot, China

**Keywords:** kaempferol, ulcerative colitis, gut microbiota, lipopolysaccharide, TLR4, NF-κB

## Abstract

Intestinal microbiota dysbiosis is an established characteristic of ulcerative colitis (UC). Regulating the gut microbiota is an attractive alternative UC treatment strategy, considering the potential adverse effects of synthetic drugs used to treat UC. Kaempferol (Kae) is an anti-inflammatory and antioxidant flavonoid derived from a variety of medicinal plants. In this study, we determined the efficacy and mechanism of action of Kae as an anti-UC agent in dextran sulfate sodium (DSS)-induced colitis mice. DSS challenge in a mouse model of UC led to weight loss, diarrhea accompanied by mucous and blood, histological abnormalities, and shortening of the colon, all of which were significantly alleviated by pretreatment with Kae. In addition, intestinal permeability was shown to improve using fluorescein isothiocyanate (FITC)–dextran administration. DSS-induced destruction of the intestinal barrier was also significantly prevented by Kae administration *via* increases in the levels of ZO-1, occludin, and claudin-1. Furthermore, Kae pretreatment decreased the levels of *IL-1β, IL-6*, and *TNF-α* and downregulated transcription of an array of inflammatory signaling molecules, while it increased *IL-10* mRNA expression. Notably, Kae reshaped the intestinal microbiome by elevating the *Firmicutes* to *Bacteroidetes* ratio; increasing the linear discriminant analysis scores of beneficial bacteria, such as *Prevotellaceae* and *Ruminococcaceae*; and reducing the richness of *Proteobacteria* in DSS-challenged mice. There was also an evident shift in the profile of fecal metabolites in the Kae treatment group. Serum LPS levels and downstream TLR4-NF-κB signaling were downregulated by Kae supplementation. Moreover, fecal microbiota transplantation from Kae-treated mice to the DSS-induced mice confirmed the effects of Kae on modulating the gut microbiota to alleviate UC. Therefore, Kae may exert protective effects against colitis mice through regulating the gut microbiota and TLR4-related signaling pathways. This study demonstrates the anti-UC effects of Kae and its potential therapeutic mechanisms, and offers novel insights into the prevention of inflammatory diseases using natural products.

## Introduction

Ulcerative colitis (UC) is the most common type of inflammatory bowel disease (IBD) and is characterized by poor prognosis, recurrent symptoms, and the potential for progression to colitis-associated cancer (1). UC is a global public health concern owing to its growing incidence (2). There are various contributors to the pathogenesis of UC, including genetic susceptibility, microbial dysbiosis, unhealthy lifestyle, and depression (3). UC is associated with defects in mucosal barrier function, leading to inflammatory cell infiltration (4). Moreover, intestinal dysbacteriosis contributes to UC pathogenesis (5). Thus, reshaping the intestinal microflora is a potential target for UC treatment intervention strategies. Clinical use of 5-aminosalicylate (5-ASA) and hormone drugs to treat UC have certain limitations, including drug dependence and severe side effects (6, 7). Consequently, there is an urgent need to develop targeted, effective, and non-toxic treatment approaches for patients with UC.

Kaempferol (Kae) is a flavonoid identified as the core active ingredient of many medicinal plants (8, 9). Kae exhibits antitumor and antioxidant properties, in addition to its ability to promote neurological recovery and regulates a variety of other biological activities (10). Further, Kae can attenuate lipopolysaccharide (LPS)-induced murine neuroinflammation by down-regulating the high mobility group protein B1/Toll-like receptor 4 (HMGB1/TLR4) pathway (11). In addition, NADPH oxidase activation and nuclear factor kappa beta (NF-κB) expression induced by advanced glycation end-products are inhibited by Kae (12). An *in vitro* study demonstrated that Kae can significantly suppress LPS/ATP-induced inflammatory responses by reducing tumor necrosis factor (TNF)-α, interleukin (IL)-6, and IL-1β release in cardiac fibroblasts (13). Moreover, a clinical study demonstrated that serum levels of C reactive protein, TNF-α, and IL-6 were decreased in type-2 diabetes patients receiving a Kae-rich diet (14). A wound healing effect of a Kae-rich diet through reduction of inflammatory mediator production, was also detected in mice with colitis (15); however, the mechanisms responsible for the link between the colitis benefits of Kae and gut microbiota are not fully understood.

Dextran sulfate sodium (DSS) has been used extensively to generate an experimental murine model of UC disease. The clinical phenotype of the animal model shares high similarity with that of patients with UC, including an increased disease activity index (DAI) (16). In addition, the model mice can exhibit typical clinicopathological features of colitis, including immune cell infiltration, mucosal barrier injury, and dysbacteriosis (17).

In this study, we investigated the effects of Kae on the interactions between the host immune system and the gut microbiota in DSS-challenged mice. Our study showed that Kae supplementation improved the intestinal barrier, restored gut microbiota, modified the metabolic profile, and suppressed the TLR4-NF-κB signaling pathway. Moreover, fecal microbial transplantation provided further validation of the potential therapeutic mechanism underlying Kae activity. Accordingly, our data suggest that Kaempferol has significant potential to alleviate UC.

## Materials and Methods

### Reagents

Kae (purity > 98%) and carboxymethyl cellulose sodium (CMC) were purchased from MeilunBio (Dalian, China). Antibodies against myeloid differentiation factor 88 (MyD88; Lot: 00091677), zonula occludens 1 (ZO-1; Lot: 10003932), occludin (Lot: 10004180) and claudin-1 (Lot: 00081584) were purchased from Proteintech (Wuhan, China). Anti-TLR4 antibody (Lot: E2913) was obtained from Santa Cruz (Santa Cruz Bio, Santa Cruz, USA). The antibody against phospho-NF-κB (*p*-NF-κB)-P65 (Lot: 16) was purchased from CST (Danvers, USA). The antibody against nucleotide oligomerization domain (NOD)-like receptor 3 (NLRP3; Lot: 080639650) was purchased from Novus Biologicals (San Diego, USA). Fluorescein isothiocyanate (FITC)-dextran (#FD4, average mol wt 3000–5000 Da) was purchased from Sigma-Aldrich (St. Louis, USA). DSS (36000–50000 Da) was obtained from MP Biochemicals (Santa Ana, USA).

### Animal Experiments

Six-week-old female C57BL/6J mice (20 ± 2g) were purchased from SPF Animals Biotechnology (Beijing, China). Mice were maintained under standard laboratory conditions, as follows: 25°C ± 3°C, 53% ± 3% humidity, with a 12-h light/dark cycle and were allowed to adapt to this environment for 7 days with free access to food and water. The composition of the mouse diet is provided in **Supplementary Table 1**. For experiments, 40 mice were randomly divided into four groups: normal control (NC), Kaempferol control (Kae), DSS-induced colitis (DSS), and Kaempferol treatment (DSS-Kae) groups (n = 10 per group). Mice in the NC and DSS groups were given vehicle (1% CMC) by gavage for 14 days, while those in the Kae and DSS-Kae groups were administered Kae (50 mg/kg/day, dissolved in 1% CMC) by gavage for 14 days. Between days 8 and 14, mice in the DSS and DSS-Kae groups received 3.5% (w/v) DSS in their drinking water.

On the 15th day of the experiment, mice were anesthetized by intraperitoneal injection with pentobarbital sodium (30 mg/kg), before samples of feces and blood were collected. At termination, all mice were euthanized by cervical dislocation. Colon samples were snap frozen in liquid nitrogen and then stored at −70°C until further study. This study was approved by the Animal Ethics Committee of Inner Mongolia Agriculture University [NO. (2020)077].

### Fecal Microbial Transplantation and Co-Housing Experiments

Fecal microbial transplantation (FMT) was achieved as described previously (18–20). Briefly, C57BL/6J female mice (n = 40; weight, 20 ± 2g) were randomly divided into four groups: F-NC, F-Kae, F-DSS, and F-DSS-Kae, and each group was designated to receive daily fresh fecal supernatant from NC, Kae, DSS, and DSS-Kae group donors, respectively. Feces from each group of mice were weighed and homogenized in saline (0.1 g/mL), the suspension centrifuged at 850 ×g, at 4°C for 5 min, and supernatants collected (>9.6 × 10^9^ CFU/mL). Recipient mice in the F-NC, F-Kae, F-DSS, and F-DSS-Kae groups were administered supernatants collected from corresponding donor mice by gavage for 14 days (10 mL/kg).The detailed co-housing experimental conditions are described in the Supplementary Material. All processes were performed under a sterile environment.

### Evaluation of Colitis

The DAI and histopathology were assessed in each group of mice. The DAI was determined by combining hematochezia, mucous stools, and body weight loss scores, and dividing them by 3 (21). Colon tissues were fixed in 4% paraformaldehyde fixation solution, dehydrated through an ethanol gradient, rendered transparent by immersion in xylene, and finally embedded in paraffin, from which 4 μm paraffin slices were sectioned and stained with hematoxylin and eosin (HE). Histological features of colon tissue samples were scored independently using a previously described method (22). Detailed scoring criteria for the DAI and histology are described in **Supplementary Tables 2** and **3**.

### Assays of Pro-Inflammatory Factor Levels

#### Enzyme-Linked Immunosorbent Assay

Serum was extracted from blood after centrifugation at 850 ×g for 20 min, 4°C. IL-1β (Lot: M200702-001a), IL-6 (Lot: M200702-004a), and TNF-α (Lot: M200702-102a) levels were measured using commercial enzyme-linked immunosorbent assay (ELISA) kits (Neobioscience Technology Co., Ltd., Shenzhen, China), following the kit instructions. Serum LPS was measured using an ELISA kit (Lot: 03036B; YaJi Biological, Shanghai, China).

#### Quantitative Reverse Transcription Polymerase Chain Reaction

TRIzol (TianGen, BeiJing, China) was used to extract total RNA from colon tissue; RNA concentration and quality were evaluated using the NanoDrop 2000C Spectrophotometer (Thermo Scientific, Waltham, USA). Prime Script RT Master Mix (TaKaRa, Beijing, China) was used to reverse-transcribe total RNA into cDNA. Transcription levels of specific genes were determined by quantitative reverse transcription polymerase chain reaction (qRT-PCR) analysis using the TB Green Premix Ex Taq II (TaKaRa, Beijing, China) and detected using the Light Cycler96 system (Roche, Mannheim, Germany). Primer sequences are shown in **Supplementary Table 4**. Relative gene expression levels were calculated by the 2^−ΔΔCT^ method.

### Immunohistochemistry

After dewaxing with xylene and an ethanol gradient, colon tissue sections were heated in sodium citrate solution for antigen retrieval, the endogenous peroxidase was inactivated by soaked in 3% hydrogen peroxide, phosphate buffered saline (PBS) was used to rinsed sections three times, and 5% normal goat serum was used to block nonspecific binding. Next, sections were incubated with primary antibodies (1:300) at 4°C overnight. After three washes with PBS, tissue sections were further incubated with biotin-tagged secondary antibodies at room temperature for 20 min. Sections were then stained with streptavidin-horseradish peroxidase, followed by the addition of the colorimetric substrate, diaminobenzidine (DAB, Beijing Solarbio Science & Technology Co., Ltd.). After staining, clearing, and dehydration, sections were sealed with gum rubber sealant. More than three visual fields were randomly selected for observation under the microscope. Quantitative analysis of the target proteins was conducted using Image J software (version 1.5.7, National Institutes of Health, USA). Protein expression intensity is expressed as positive protein integral optical density.

### 16S rRNA Gene Sequencing

Fecal genomic DNA was extracted by DNeasy-PowerSoil Kit (Qiagen, Dusseldorf, Germany) following the standard procedure. DNA concentrations were determined using the NanoDrop 2000C Spectrophotometer and gel electrophoresis. Using genomic DNA as the template, specific primers (343F 5’-TACGGRAGGCAGCAG-3’ and 798R 5’-AGGGTATCTAATCCT-3’) with barcodes and Tks Gflex DNA Polymerase (TaKaRa, Beijing, China) were used to amplify the V3-V4 hypervariable regions of the bacterial 16S rRNA gene. The quantity and quality of amplicons were evaluated using the NanoDrop 2000C Spectrophotometer and gel electrophoresis. Subsequently, AMPure XP beads were used to purify the amplicons (Beckman Coulter, California, USA) and purified products formed the template for the next round of amplification. The quality of amplicons was again confirmed as described above, and products were purified once more using AMPure XP beads. After the second purification step, the Qubit-dsDNA analysis kit (Life Technologies, Waltham, USA) was used to quantify the amplicons. Pooled equal amounts of purified amplicons were sequenced on the Illumina Novaseq platform (Illumina, California, USA) to obtain raw data in FASTQ format.

### GC-MS Untargeted Metabolomics

Mice feces (50 mg) were transferred into a 2 mL microcentrifuge tube, and mixed with 500 μL of extraction solvent (methanol/water 4:1 ratio, v/v), 40 μL of internal standard solution (2-chloro-l-phenylalanine in methanol, 3 g/L), and ground at 60 Hz for 3 min. Samples were then added to 120 μL of chloroform and subjected to vigorous vortexing, followed by ultrasonic extraction for 20 min at 25°C. Supernatants were extracted after samples were centrifuged at 13,680 ×g, for 20 min at 4°C, dried at 25°C under vacuum for 30 min, and dissolved in 80 μL of 15 mg/mL methoxyamine hydrochloride in pyridine. The resulting products was vigorously vortexed for 10 min, and kept at room temperature for 80 min. To this mixture, 20 μL of n-hexane and 60 μL of BSTFA (containing 2% TMCS) were added, samples vortexed vigorously for 3 min, then derivatized at 65°C for 70 min, and analyzed by gas chromatography using the Agilent 7890 B System, coupled to the Agilent 5977 A MSD System (Agilent Technology, California, USA). Separation of derivatives was performed using a 30 mm× 0.25 mm× 0.25 µm DB-5MS fused silica capillary column (Agilent Technology, California, USA), using the standard protocol, under full-scan mode to detect mass spectrometry data (m/z 50–500). Quality control samples were injected regularly throughout the analysis process to check data reproducibility. Raw data were obtained in D format.

### Bioinformatics Analysis

FASTQ format raw data were processed using Trimmomatic (version 0.39) and FLASH (version 1.2.11), to remove invalid bases (23, 24). Data were classified into multiple operational taxonomic units (OTUs) by Vsearch (version 2.15.1) according to similarity score (≥ 97%) (25). Representative sequences of every OTU were selected using QIIME software and compared with the Greengenes database (v201305) (26). Alpha-diversity were assessed using the Shannon, Simpson, and Chao1 indices in Mothur (version 1.44.3) and R language (27). Pairwise comparisons were analyzed by Wilcoxon rank sum test for alpha-diversity. To evaluate beta-diversity, principal component analysis (PCA), principal coordinates analysis (PCoA), and non-metric multidimensional scaling (NMDS) were computed using QIIME and R language. Galaxy LEfSe tools were used to conduct linear discriminant analysis effect size (LEfSe) analyses and the relative abundance of microbial taxa and (LDA) scores >2 were recorded (28). Different microbial taxa were distinguished by the non-parametric factorial Kruskal-Wallis sum-rank test. Raw metabolomic data were quality-filtered using Analysis Base File Converter (version 1.0), then representative data was searched against the untargeted GC-MS database (OEbio, Shanghai, China). After log10 transformation, partial least-squares discrimination analysis (PLS-DA) and orthogonal partial least-squares-discriminant analysis (OPLS-DA) were performed using a data matrix to visualize the metabolic differences between the DSS and DSS-Kae groups. Differential metabolites were analyzed by calculation of Pearson correlation coefficients. Kyoto encyclopedia of genes and genomes (KEGG) pathway enrichment analysis was conducted using tools available in the KEGG database (release 95.2) (www.kegg.jp).

### Measurement of Intestinal Permeability

Intestinal permeability was evaluated using a FITC-labelled-dextran method as described by Volynets et al. (29). Briefly, on the final day of the experiment, FITC–dextran (mol wt 3000–5000 kDa; 600 mg/kg) was administered to the mice by oral gavage 4 h before euthanization. Immediately before euthanization, blood was collected and heparinized. Plasma was separated by centrifugation at 12000 ×g for 10 min, at 4°C, and 200 µL of each sample was added to a 96-well black microplate. Fluorescence was read with Agilent Biotek Synergy H4 (Santa Clara, USA) at 485/528 nm wavelength.

### Statistical Analysis

Statistical analyses were performed using SPSS 22.0 software (New York, USA). Data are expressed as mean ± standard error of the mean (SEM). One-way analysis of variance (ANOVA) followed by Tukey *post hoc* analysis was applied when evaluating differences between two groups. *P* values < 0.05 were considered statistically significant.

## Results

### Kae Attenuated DSS-Induced Murine Colitis

**Figure 1:**
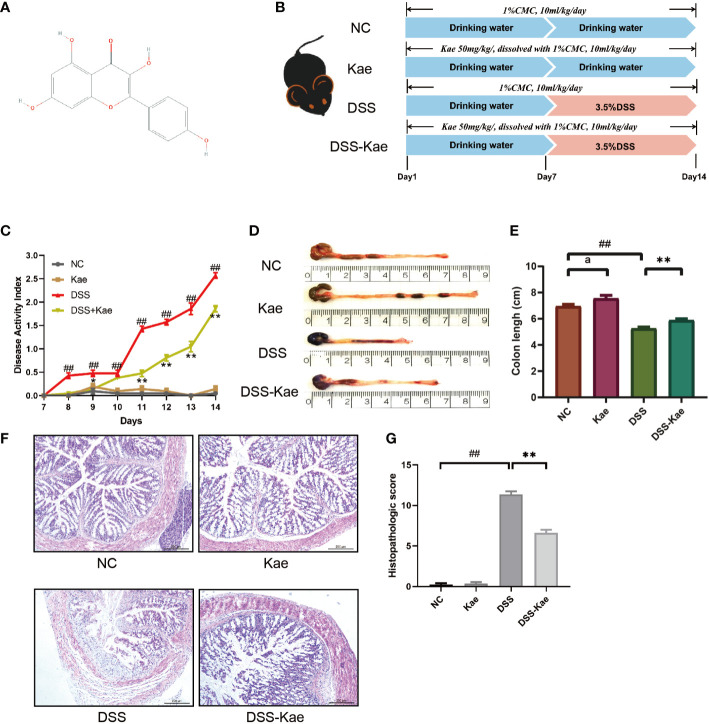
Kae attenuates the symptoms of DSS-induced mice colitis. **(A)** Chemical structure of kaempferol (Kae). **(B)** Experimental design to test the effects of Kae on DSS-induced mice (n = 10/group). **(C)** Disease activity index of mice during the course of colitis. **(D, E)** Representative images of colons from mice following euthanization and statistical analysis of colon length in each group. **(F, G)** Representative images of HE stained colon tissue samples (scale bar, 200 μm) and histological scores of colonic tissues. Data are expressed as the mean ± SEM, n = 10, analyzed using one-way ANOVA with Tukey post-hoc analysis. DSS (*vs*. NC, ^##^ *P* < 0.01; *vs*. DSS-Kae, *P < 0.05, **P < 0.01); Kae (*vs*. NC, ^a^ *P* < 0.05).

The structure of Kae is shown in **Figure 1A**. According to the experimental treatment schedule (**Figure 1B**), mice in the DSS and DSS-Kae groups were supplied *ad libitum* with 3.5% (w/v) DSS in drinking water on days 7–14, while those in the NC and Kae groups had *ad libitum* access to untreated drinking water. Compared with the NC group, the DSS group had an obviously steeper DAI score curve, while the rise in the DSS-Kae group was slower (*P <* 0.05) (**Figure 1C**). Additionally, treatment with Kae led to an increased colon length, relative to the DSS group (*P <* 0.05) (**Figures 1D, E** **)**. HE staining of colon tissue showed that DSS treatment caused severe enteric mucosal injury; however, all characteristic features were prevented by oral Kae supplementation (**Figures 1F, G** **)**. These results indicated that Kae could alleviate the gross symptoms of DSS-induced colitis in mice and alleviated colonic injury.

### Kae Attenuated DSS-Triggered Pro-Inflammatory Responses

**Figure 2:**
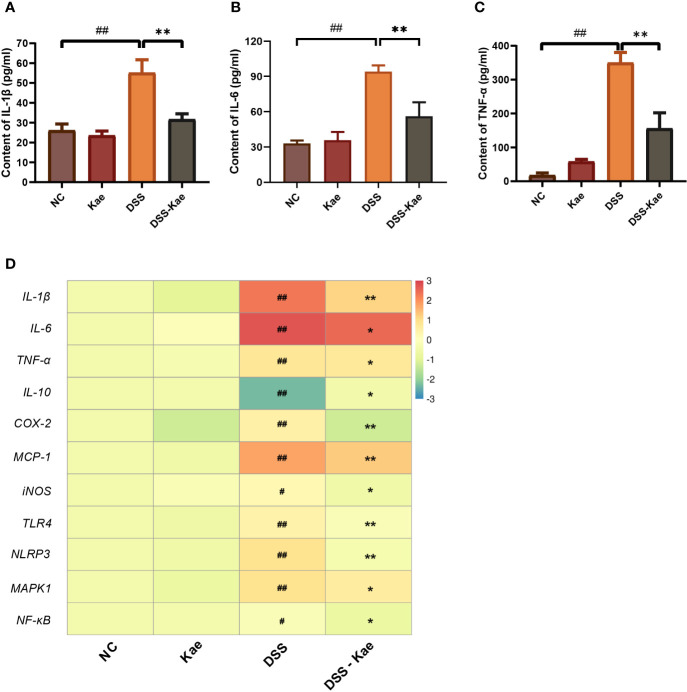
Effects of Kae on inflammatory-associated cytokine levels in DSS- and Kae-treated mice. Serum inflammatory factors, **(A)** IL-1β, **(B)** IL-6, and **(C)** TNF-α, detected using ELISA kits. **(D)** Relative mRNA expression of inflammatory factors in the colon evaluated by qRT-PCR. All data were log_2_ converted and are presented as fold-change in expression level versus the NC group (means for the NC group were set as 1). Data are expressed as the mean ± SEM, n = 4–6, analyzed using one-way ANOVA with Tukey post-hoc analysis. DSS (*vs*. NC, ^#^ *P* < 0.05, ^##^ *P* < 0.01; *vs*. DSS-Kae, *P < 0.05, **P < 0.01).

Next, we assessed the effects of Kae administration on DSS-induced pro-inflammatory responses. Compared with the NC group, DSS triggered significantly increased serum IL-1, IL-6, and TNF-α levels. Conversely, Kae supplementation remarkably reversed this tendency (**Figures 2A–C**). DSS treatment significantly increased mRNA level expression of inflammatory factors, such as *IL-1β*, *IL-6*, *TNF-α*, *COX-2*, *MCP-1*, and *iNOS*, while inhibiting *IL-10* expression (*P <* 0.05) (**Figure 2D**). Further, mRNA expression levels of the pattern recognition receptors, *TLR4* and *NLRP3*, as well as the signaling molecules, *MAPK1* and *NF-κB*, were also elevated in the DSS group. In contrast, gavage with Kae strongly inhibited the transcription of genes involved in colitis, while reversing the effects of DSS on *IL-10* transcription (*P <* 0.05). In summary, Kae alleviated inflammation caused by UC, partly through upregulation of *IL-10* transcription and downregulation of the expression of inflammation-associated genes.

### Kae Prevented Loss of Intestinal Barrier Integrity in Mice Treated With DSS

**Figure 3:**
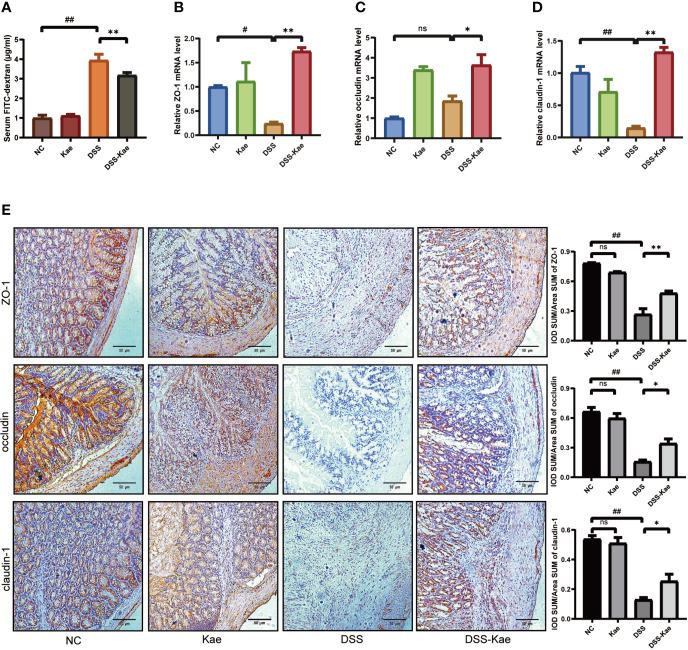
Kae improves gut permeability and enhances expression of intestinal tight junction proteins. Relative mRNA levels analysis of **(A)** Quantification of serum FITC-dextran. **(B)** *ZO-1*, **(C)** *occludin*, and **(D)** *claudin-1*. **(E)** Representative images of immunohistochemical staining of ZO-1, occludin, and claudin-1 in colon samples from different experimental groups (scale bar, 50 μm). Positive protein integral optical density was determined using Image J 1.5.7 software. Data are expressed as the mean ± SEM, n = 5–6, analyzed using one-way ANOVA with Tukey post-hoc analysis. DSS (*vs*. NC, ^#^ *P* < 0.05, ^##^ *P* < 0.01; *vs*. DSS-Kae, *P < 0.05, **P < 0.01); ns, no significant difference.

To evaluate the protective effects of Kae administration on intestinal barrier integrity, fluorescence spectroscopy of ingested FITC–dextran was measure. qRT-PCR and immunohistochemistry were used to evaluate the expression levels of tight junctions (TJs) genes, including ZO-1, occludin, and claudin-1. The results showed that mice with DSS exposure had remarkably higher FITC levels, Kae pre-treatment revealed an improvement in intestinal permeability compared to DSS-only mice (*P <* 0.05) (**Figure 3A**). DSS challenge led to decreased levels of ZO-1, occludin, and claudin-1, whereas in mice pretreated with Kae, expression of ZO-1, occludin, and claudin-1 were similar to those of control untreated mice (**Figures 3B–E**). These data suggested that the anti-inflammation effects of Kae involved maintenance of intestinal barriers.

### Kae Reshaped the Diversity and Richness of the Gut Microbiota

**Figure 4:**
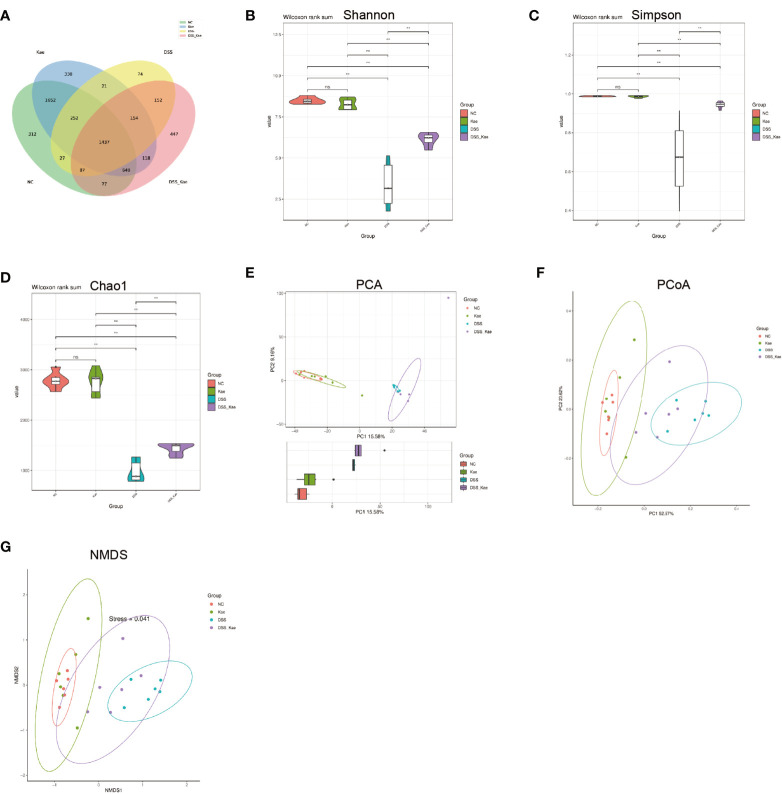
Kae increased the diversity and richness of gut microbiota in DSS-induced mice. **(A)** Venn diagram illustrates the numbers of OTUs in the NC, Kae, DSS, and DSS-Kae groups. Alpha diversity is illustrated using a violin plot of the **(B)** Shannon, **(C)** Simpson, and **(D)** Chao indices. Beta-diversity was assessed using **(E)** PCA, **(F)** PCoA, and **(G)** NMDS, based on weighted UniFrac distances. Pairwise comparisons using the Wilcoxon rank sum test for alpha diversity, n = 6, **P < 0.01; ns, no significant difference.

In this study, we performed 16S rRNA gene high-throughput sequencing to reveal the impact of Kae on the gut microbiota. Results from rarefaction curve analysis, Shannon index calculation, and Good’s coverage reflected that there was a sufficient sequencing depth to provide coverage of the majority of microflora diversity in each sample (**Supplementary Figure S1**). Generation of a Venn diagram showed that in all samples, total of 1473 OTUs were calculated, 312 and 300 were in the NC and Kae groups, respectively, while the DSS group had fewer OTUs (n = 74), compared with the DSS-Kae groups (n = 447) (**Figure 4A**).

Alpha-diversity analysis is mainly used to assess community diversity and richness. Compared with samples from the NC and Kae groups, samples from the DSS group had remarkably reduced Shannon and Simpson diversity indices, and a lower Chao1 richness estimates, whilst Kae treatment effectively prevented the DSS-induced decline in bacterial community diversity and richness (*P <* 0.05) (**Figures 4B–D**).

PCA, PCoA, and NMDS analyses were used to evaluate similarities and differences between samples and groups. Based on values generated using the weighted UniFrac algorithm, PCoA and NMDS analyses indicated that the gut microbiota composition of the NC group was unlike that of the DSS group in terms of Axis PCo-1 and NMDS-1. Moreover, the DSS-Kae group was clearly located on a separate branch from the DSS group, but close to the NC and Kae groups (**Figures 4E–G**). These results indicated that the gut microbiota community structure was maintained by administration of Kae.

### Kae Administration Restructured the Gut Microbiota Diversity in Mice Treated With DSS

**Figure 5:**
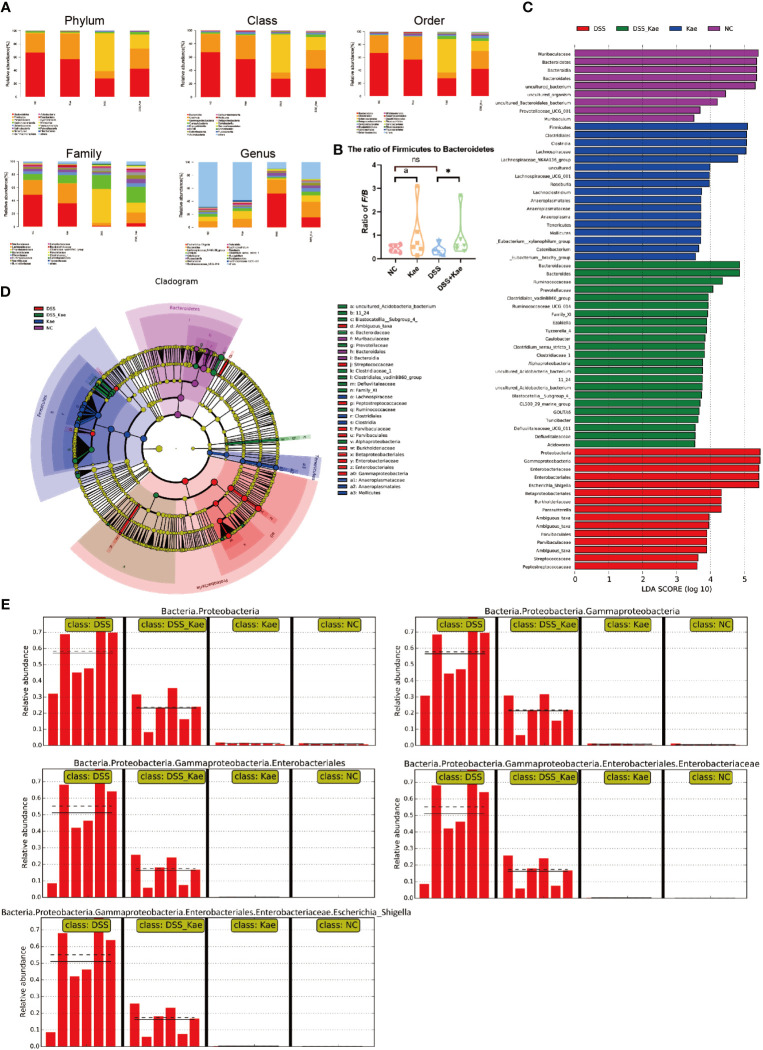
**(A)** Taxonomic analysis of microbiota in fecal samples at the phylum, class, order, family, and genus levels. **(B)** Ratio of *Firmicutes* to *Bacteroidetes* in the gut microbiota. **(C)** LDA scores for bacterial taxa significantly enriched in gut microbiota from each group (LDA score > 3). **(D)** Cladogram illustrating the results of LEfSe analysis. **(E)** All-against-all algorithm of LDA coupled with LEfSe. Ratios are expressed as the mean ± SEM, n = 6, analyzed using one-way ANOVA with Tukey post-hoc analysis. DSS (*vs*. DSS-Kae, *P < 0.05); Kae (*vs.* NC, ^a^ *P* < 0.05); ns, no significant difference. The significance of differences in taxonomic groups were assessed using the non-parametric factorial Kruskal-Wallis sum-rank test, n = 6. P < 0.05 was considered to indicate a significant difference between groups.

As shown in **Figure 5A**, DSS challenge resulted in an obvious decrease in the abundance of *Bacteroidetes* and *Firmicutes*, while the abundance of *Proteobacteria* was increased. Kae supplementation prevented the decrease in *Firmicutes*/*Bacteroidetes* ratio (**Figure 5B**). LEfSe analysis showed that pathogenic bacteria, such as *Proteobacteria, Gammaproteobacteria*, and *Enterobacteriaceae*, had LDA scores >4 in the DSS group. On the contrary, the DSS-Kae group had higher scores for beneficial bacteria, such as *Ruminococcaceae* and *Prevotellaceae* (**Figures 5C, D**). The all-against-all algorithm within LEfSe demonstrated that, in the DSS group, *Proteobacteria*, *Gammaproteobacteria*, *Enterobacteriales*, *Enterobacteriaceae*, and *Escherichia_Shigella* species were remarkably increased, whereas Kae supplementation partially prevented increases in these bacteria (**Figure 5E**).

### Protective Effects of Kae Treatment on Gut Microbiota-Derived Metabolites

**Figure 6:**
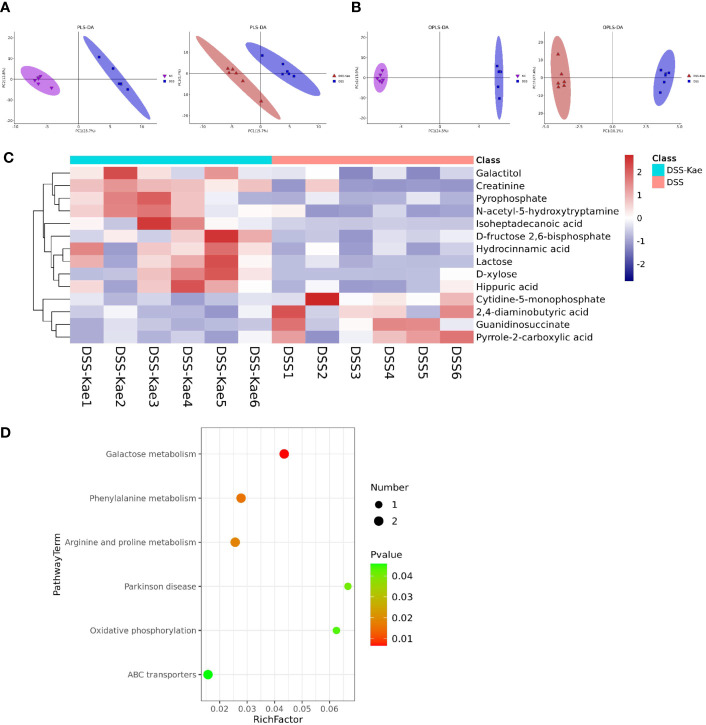
Kae altered fecal metabolic composition. **(A)** PLS-DA analysis score plot showing comparisons of the NC *vs* DSS and DSS *vs* DSS-Kae group metabolome profiles. **(B)** OPLS-DA analysis score plots showing comparisons of the NC *vs* DSS and DSS *vs* DSS-Kae group metabolome profiles. **(C)** Heat map showing metabolites differing significantly in abundance between the DSS and DSS-Kae groups. **(D)** KEGG pathway analysis of the DSS and DSS-Kae groups. Statistical analysis was conducted by calculation of Pearson correlation coefficients (VI*P* > 1 and *P* < 0.05).

We performed untargeted metabolomics analysis to study the effect of Kae supplementation on the metabolic profiles of mice with colitis. PLS-DA and OPLS-DA analyses showed clearly distinguished between the metabolic profiles of mice in the DSS and DSS-Kae groups (**Figures 6A, B**), and demonstrated that the DSS-treated group formed a distinct metabolic cluster, separate from those of the NC and DSS-Kae groups. In addition, 14 metabolites showing differential abundance between the DSS and DSS-Kae groups were identified. Interestingly, we found that supplementation with Kae greatly increased the levels of D-fructose 2,6-bisphosphate, D-xylose, galactitol, lactose, and N-acetyl-5-hydroxytryptamine in DSS-treated mice (**Figure 6C**). Pathway enrichment analysis indicated that Kae elicited major alterations in metabolic pathways related to phenylalanine metabolism, galactose metabolism, and arginine and proline metabolism (**Figure 6D**). Taken together, these results demonstrated that the protective effects of Kae against UC were related to the regulation of microbial metabolites.

### Down-Regulation of LPS-TLR4-NF-κB Inflammatory Pathway by Kae

**Figure 7:**
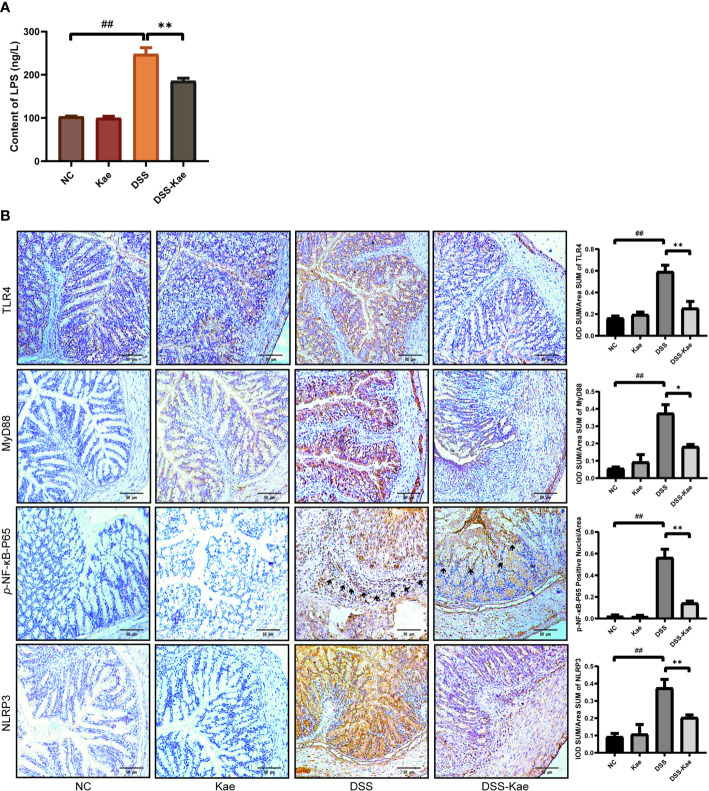
Kae suppresses the LPS-TLR4-NF-κB signaling pathway. **(A)** Serum levels of LPS. **(B)** Immunohistochemical analysis of TLR4, MyD88, *p*-NF-κB-P65, and NLRP3 expression in colon tissues (scale bar, 50 μm). Positive protein integral optical density was analyzed using Image J 1.5.7 software. Data are expressed as mean ± SEM, n = 5, analyzed using one-way ANOVA with Tukey post-hoc analysis. DSS (*vs*. NC, ^##^ *P* < 0.01; *vs*. DSS-Kae, *P < 0.05, **P < 0.01), ns, no significant difference.

As shown in **Figure 7A**, the LPS content in serum was significantly increased in the DSS group, indicating that DSS triggered metabolic endotoxemia. Conversely, the level of LPS in the DSS-Kae group was remarkably lower, suggesting that Kae was able to alleviate DSS-induced endotoxemia. Immunohistochemistry analysis showed that Kae pretreatment inhibited DSS-induced expression of TLR4, MyD88, *p*-NF-κB-P65, and NLRP3 (**Figure 7B**). Therefore, the anti-colitis activity of Kae could be partly attributed to the inhibition of the LPS-TLR4-NF-κB inflammatory pathway.

### Anti-Colitis Effects of Kae Could Be Induced by Microbiota-Transfer

**Figure 8:**
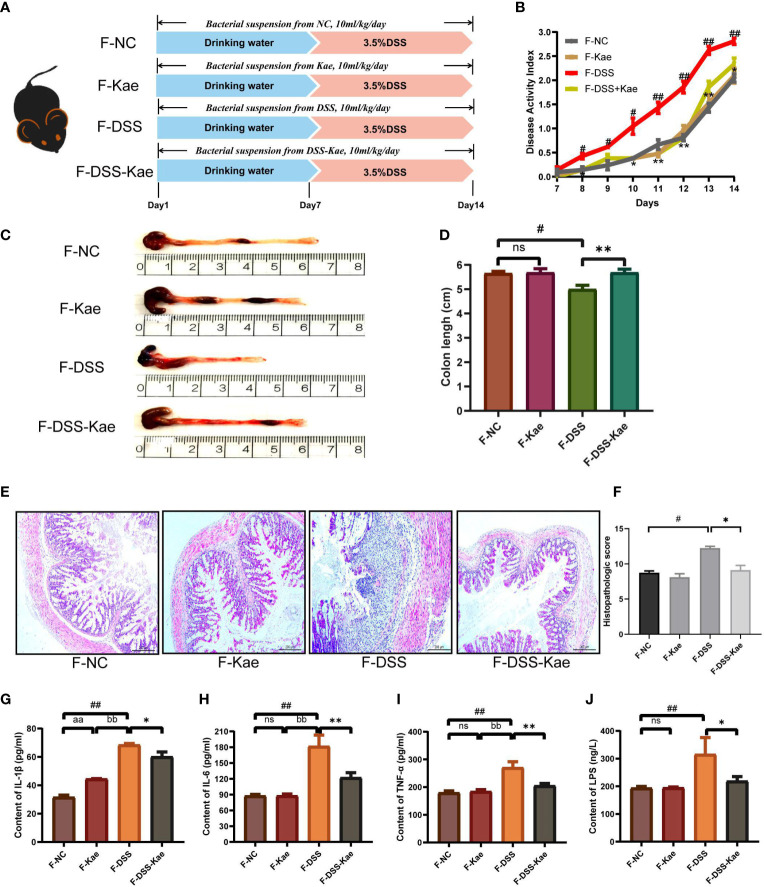
Transplantation of microbiota altered in response to Kae recapitulates the effects of Kae treatment on DSS-induced colitis. **(A)** Design of the FMT experiment on DSS-treated mice (n = 10/group). **(B)** Disease activity index of FMT mice during the course of colitis. **(C, D)** Representative images of mouse colon at sacrifice and statistical analysis of colon length data from each FMT group. **(E, F)** Representative images of H&E staining of colon samples (scale bar, 200 μm) and histological scores of colonic tissues. Serum inflammatory factors: **(G)** IL-1β, **(H)** IL-6, and **(I)** TNF-α were measured using ELISA kits. **(J)** Serum LPS levels indicate the endotoxemia index. Data are expressed as the mean ± SEM, n = 4–10, analyzed using one-way ANOVA with Tukey post-hoc analysis. DSS (*vs*. NC, ^#^ *P* < 0.05; ^##^ *P* < 0.01; *vs*. DSS-Kae, *P < 0.05, **P < 0.01); F-Kae (*vs*. F-NC, ^aa^ *P* < 0.01); F-Kae (*vs*. F-DSS, ^bb^ *P* < 0.01); ns, no significant difference.

To further assess the involvement of the gut microbiota and its metabolic products in the anti-inflammatory effects of Kae, FMT and co-housing experiments were established (**Figure 8A**, **Supplementary Figure S2A**). The results showed that FMT from DSS-Kae donor mice to DSS-challenged recipient mice significantly increased colon length, reduced DAI scores, and alleviated pathological features (**Figures 8B–F**). Further, serum IL-1β, IL-6, TNF-α, and LPS content were also decreased in the recipient group (*P <* 0.05) (**Figures 8G–J**). The results of co-housing experiments showed that the anti-colitis effects of Kae could be transferred among co-housed mice to some extent (**Supplementary Figures S2B-I**). These results further verified the beneficial effects of Kae against colitis were associated with its ability to regulate the microbiota.

**Figure 9:**
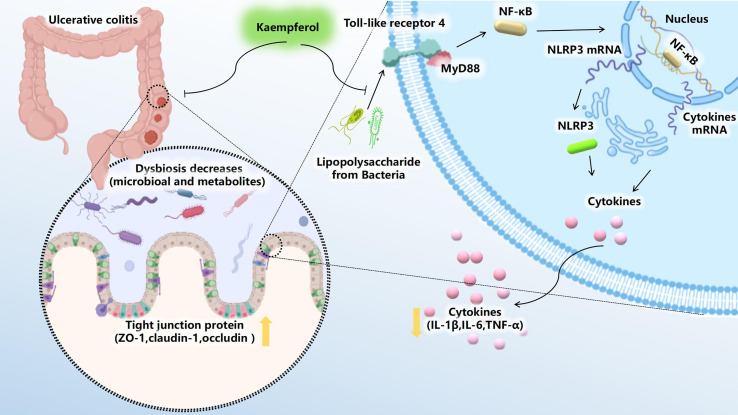
Kae exerts excellent anti-UC effects *via* gut microbiota pathways related to the LPS-TLR4-NF-κB core pathway. Kae reduces LPS levels by inhibiting the proliferation of pathogenic Gram-negative bacilli, thereby altering the metabolic profile, blocking NF-κB pathway activation, improving intestinal tight junction integrity, inhibiting pro-inflammatory factors, and increasing antioxidants, thus decreasing DSS-induced colonic inflammation.

## Discussion

UC is an idiopathic IBD which is incurable and prone to relapse (30, 31). Research to date indicates that IBD is a “polymicrobial disease”, involving a combination of interactions between microorganisms and the host (32). Modulation of the gut microbiota is a new therapeutic option for UC patients. Nutritional therapies, including exclusive enteral nutrition, have already been demonstrated to be clearly linked with changes in the gut microbiota in colitis patients (33, 34). In our studies, we used DSS to induce UC in model mice, since it can successfully induce high rates of stable colitis-associated characteristics (35). As expected, mice with DSS-induced colitis showed a variety of disease features comparable to those observed in patients with UC, including increased DAI score and intestinal micro-dysbiosis.

The anti-UC potential of Kae has been used to guide the management of many clinical conditions (36). Bian et al. (37) used a coculture model of gut endothelial and epithelial cells to study the effects of Kae, and their results indicated that Kae could alleviate epithelial barrier dysfunction and inhibit IL-8 secretion *via* suppressing NF-κB-related pathways; however, there is the insufficient data *in vivo* to support these *in vitro* results. Another study demonstrated the beneficial effects of Kae against colitis (15), although the potential role of the intestinal microbiome in mediating the beneficial effects of Kae was not elucidated. The current study aimed to further unravel the mechanisms underlying the effects of Kae on gut microbiota and then influence colonic inflammation in a DSS-triggered UC mice model. Our results suggested that Kae supplementation alleviates UC in this mice model through modulation of the intestinal microbiota and TLR4-related pathways.

Gut microbiota are an essential part of the intestinal barrier, and have critical roles in host pathophysiological processes, such as intestinal mucosal barrier maturity, immune system development, nutrient absorption, and energy metabolism (38). Increasing evidence indicates that a decline in the diversity and richness of intestinal microbiota is correlated with an increased prevalence of colitis (39). The major phyla constituting intestinal microorganisms include *Firmicutes* and *Bacteroides* (40). One clinical study demonstrated that the *Firmicutes* to *Bacteroides* ratio (F/B) decreased in UC patients (41), while the abundance of *Proteobacteria* increased significantly in mice with colitis, and was also a signature feature of gut dysbiosis (42). Our study showed that Kae could reverse the F/B ratio in DSS-treated mice. Interestingly, Kae pretreatment reshaped the microbiota composition by decreasing the abundance of *Proteobacteria*. Moreover, the probiotic *Prevotellaceae* and *Ruminococcaceae* phyla increased in response to Kae administration. Chen et al. pointed out that the hydrogen-producing *Prevotellaceae* can be considered antioxidant organisms that can neutralize reactive oxygen species, protecting cells against oxidative stress and alleviating symptoms of IBD in patients (43). *Ruminococcaceae* are an important type of butyrate-producing bacteria. A recent study has shown that DSS challenge results in a lower relative abundance of *Ruminococcaceae* in both the cecum and small intestine (44). Recent research by Zhang et al. demonstrated that *Dendrobium officinale* polysaccharides can ameliorate the extent of colitis by increasing the abundance of *Ruminococcaceae*, a type of short-chain fatty acid (SCFA)-producing bacteria (45). Our study highlights increased diversity in commensal bacterial when mice were administered with DSS following Kae pretreatment. Future research will be imperative to decipher whether SCFA plays a role in the anti-UC efficacy of Kae.

During digestion, the gut microbiota produces large quantities of metabolites. These metabolites interact with intestinal epithelial cells, can enter the circulatory system, and have important functions in human health and disease (46). Galactose metabolism is involved in maintaining the energy intake of cells, whereas metabolism of phenylalanine, arginine, and proline is strongly associated with several human diseases (47–49). FMT has been widely considered as an effective strategy to re-establish an intestinal ecosystem (50). Using FMT, we further demonstrated that the intestinal microflora plays a critical role in regulating the influence of Kae on UC mice. Coprophagy is the common habit that rodents feed on each other’s feces, and the behavior will meet their own nutritional needs, maintain the stability of gut microbiota, and help rodents to maintain a normal level of memory and cognition (51). Through fecal-oral transplantation of intestinal microorganisms, our data indicate that the anti-colitis effects of Kae can be transferred among co-housed mice, implying the involvement of the gut microbiota in the mechanism of action of Kae. Longer co-housed experimental observation time is necessary in the future in order to fully evaluate the effects of Kae.

Sequencing of microbial 16S rRNA genes revealed that *Proteobacteria* was pivotal in the processes investigated in our study. Increases in the abundance of gram-negative bacteria are more closely-linked with UC progression (42). Initial recognition of *Proteobacteria* in the intestinal tract occurs through pathogen recognition receptors, of which TLR4 is a specific receptor for the LPS released from *Proteobacteria* (52). Upon ligand binding, MyD88-dependent signaling can result in the phosphorylation of NF-κB, a transcription factor that regulates the levels of IL-1β, IL-6, TNF-α, and several other inflammatory factors, in response to NLRP3 (53, 54). Consistent with previous investigations (55, 56), our study showed that Kae could inhibit the expression of inflammatory-associated mediators by dampening the activation of the LPS-TLR4-NF-κB signaling in mice with DSS-triggered colitis.

Intestinal epithelial barrier dysfunction is a fundamental component of UC pathogenesis (57). The damage of the intestinal mucosal barrier is the initiating factor of colitis, and can lead to elevated intestinal permeability and infiltration of antigens, toxins, and pathogens from the intraluminal environment into the mucosal tissue, leading to the onset of inflammation (58). The FITC-dextran test has already been used successfully in previous studies to assess intestinal permeability in mice induced with DSS (59). The integrity of TJs determines the permeability of the intestine; thus, TJs are of great importance in determining the integrity of the intestinal epithelial barrier (60); their “zipper-like” structure can effectively close the intercellular space and prevent the infiltration of harmful substances (58). TJs mainly consist of transmembrane proteins, including the cytoplasmic proteins, ZO-1, occludin, and claudins (61). Occludin have the c-terminal coiled-coil domain, binds with ZO-1 to regulate intercellular signaling and message transmission, and affects actin contractility to control colonic permeability (62). Claudin, the main skeletal protein of TJs, is widely expressed in the basement membrane (63). Analysis of FITC revealed that Kae reduced the intestinal permeability compared to the DSS group. Furthermore, we demonstrated that Kae could markedly restore the expression of ZO-1, occludin, and claudin-1 in the murine colitis model, likely recovering the integrity of the intestinal mucosal barrier.

In conclusion, our study demonstrated that Kae exerts immunoregulatory effects in mice with UC by regulation of the gut microbiota and a wide range of metabolites, thereby suppressing LPS-induced TLR4-NF-κB signaling. Intestinal dysbiosis is associated with a variety of pathological processes including UC. Understanding this relationship is crucial to the full exploitation of the therapeutic potential of microbial interventions in clinical treatment. Therefore, this investigation provides a novel insight that foods rich in Kae may have a health benefit for preventing human UC and more broadly reveals metabolic processes involving intestinal microbiota.

## Data Availability Statement

The datasets presented in this study can be found in online repositories. The names of the repository/repositories and accession number(s) can be found below: https://www.ncbi.nlm.nih.gov/, PRJNA707141.

## Ethics Statement

The animal study was reviewed and approved by Animal Ethics Committee of Inner Agriculture Medical University.

## Author Contributions

YQ and YW conceived the experiments. XL and FX designed and performed the animal studies. XW and YQ performed the molecular biology experiments. SZ and JX provided the technical support. YQ and YW drafted and edited the manuscript. All authors contributed to the article and approved the submitted version.

## Funding

This research was supported by Grants from the Natural Science Foundation of Inner Mongolia (2020MS08055, 2019MS03019) and the program for Yong Talents of Science and Technology in Universities of Inner Mongolia Autonomous Region (No. NJYT-19-A14).

## Conflict of Interest

The authors declare that the research was conducted in the absence of any commercial or financial relationships that could be construed as a potential conflict of interest.

## Abbreviations

5-ASA, 5-aminosalicylate; ANOVA, analysis of variance; CMC, carboxymethyl cellulose sodium; DAI, disease activity index; DSS, dextran sulfate sodium; ELISA, enzyme linked immunosorbent assay; fluorescein isothiocyanate, FITC; FMT, fecal microbial transplantation; HE, hematoxylin–eosin; HMGB1, high mobility group protein B1; IBD, inflammatory bowel disease; IL, interleukin; Kae, Kaempferol; KEGG, Kyoto encyclopedia of genes and genomes; LDA, linear discriminant analysis; LEfSe, linear discriminant analysis effect size; LPS, lipopolysaccharide; MyD88, myeloid differentiation factor 88; NC, normal control; NF-κB, nuclear factor kappa-B; NLR, NOD-like receptor; NOD, nucleotide oligomerization domain; OPLS-DA, orthogonal partial least-squares-discriminant analysis; OTUs, operational taxonomic units; PBS, phosphate buffer saline; PCA, principal component analysis; PCoA, principal coordinates analysis; PLS-DA, partial least-squares discrimination analysis; qRT-PCR, quantitative reverse transcription polymerase chain reaction; SCFA, short-chain fatty acid; SEM, standard error of the mean; TJ, tight junction; TLR4, toll-like receptor 4; TNF-α, tumor necrosis factor-α; UC, ulcerative colitis; ZO-1, zonula occludens 1.
