# Supplementary material for: Kaempferol Alleviates Murine Experimental Colitis by Restoring Gut Microbiota and Inhibiting the LPS-TLR4-NF-κB Axis
Source: Front Immunol. 2021 Jul 22;12:679897. doi: 10.3389/fimmu.2021.679897 (PMC8339999; doi:10.3389/fimmu.2021.679897)
Supplement: Supplementary file 1 [file DataSheet_1.docx]

Supplementary Material

# Assessment of 16s rRNA gene sequencing coverage

Rarefaction curves were used to evaluate whether sequencing coverage was sufficient to optimally cover all taxonomic groups; if the sequencing is sufficiently deep, curves will reach a plateau. Our results show that the rarefaction curves for the Shannon index and Good’s coverage reached plateau **(Supplementary Figure S1)**, indicating adequate sequencing depth and the capture of the majority of microbial diversity in the samples.


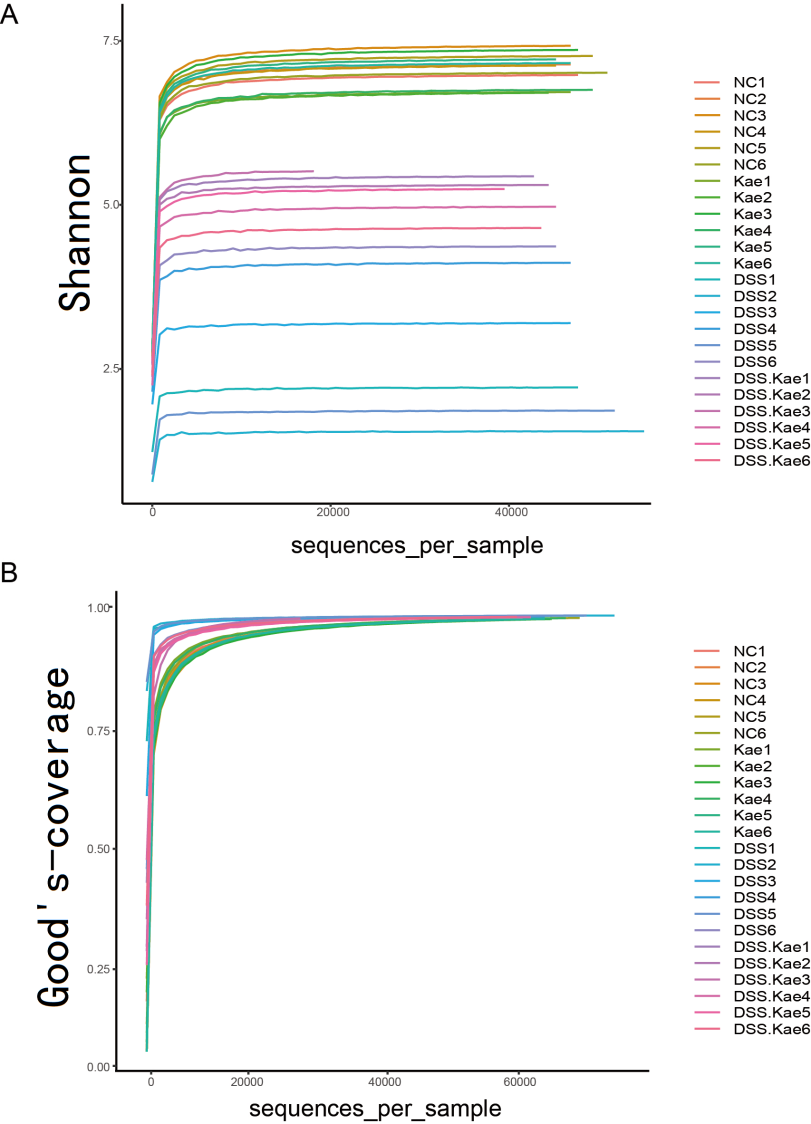


**Supplementary Figure S1.** Rarefaction curves for (A) the Shannon index and (B) Good’s coverage.

# Co-housing with Kae-treated mice ameliorated DSS-induced colitis

To further understand the impact of Kae on the gut microbiome, we conducted microbiota-transfer studies by co-housing mice receiving different treatments. Mice have particular fecal feeding behavior; they practice gastrointestinal auto- and allo-reinoculation of large intestine microbiota, via fecal ingestion (or coprophagy) in laboratory settings. Using this characteristic, we investigated whether the curative effects of Kae and its regulation of the gut microbiome could be mediated via the fecal-oral route. The DSS and DSS-Kae groups were renamed the Sh (single-housing)-DSS and Sh-DSS-Kae groups. Six-week-old female C57BL/6J mice (n = 14) were randomly divided into two groups: co-housing (Ch)-DSS and Ch-DSS-Kae, which lived in the same cage, but received different treatments. The Ch-DSS group were administered 1% CMC (dissolved in ddH_2_O) by oral gavage for 14 days while the Ch-DSS-Kae group received Kae (50 mg/kg/day, dissolved with 1% CMC with ddH_2_O) by oral gavage for 14 days. On days 8–14, mice were supplied with drinking water containing 3.5% (w/v) DSS. As expected, Ch-DSS mice had longer colons, lower DAI scores, and healthier histological features **(Supplementary Figure S2)**. Hence, living together with Ch-DSS mice did not appear to affect the efficacy of Kae in reducing inflammation. Therefore, our data indicated that the anti-inflammatory effects of Kae can be transferred among co-housed mice, implicating the involvement of the gut microbiota in the mechanism of action of Kae.

**
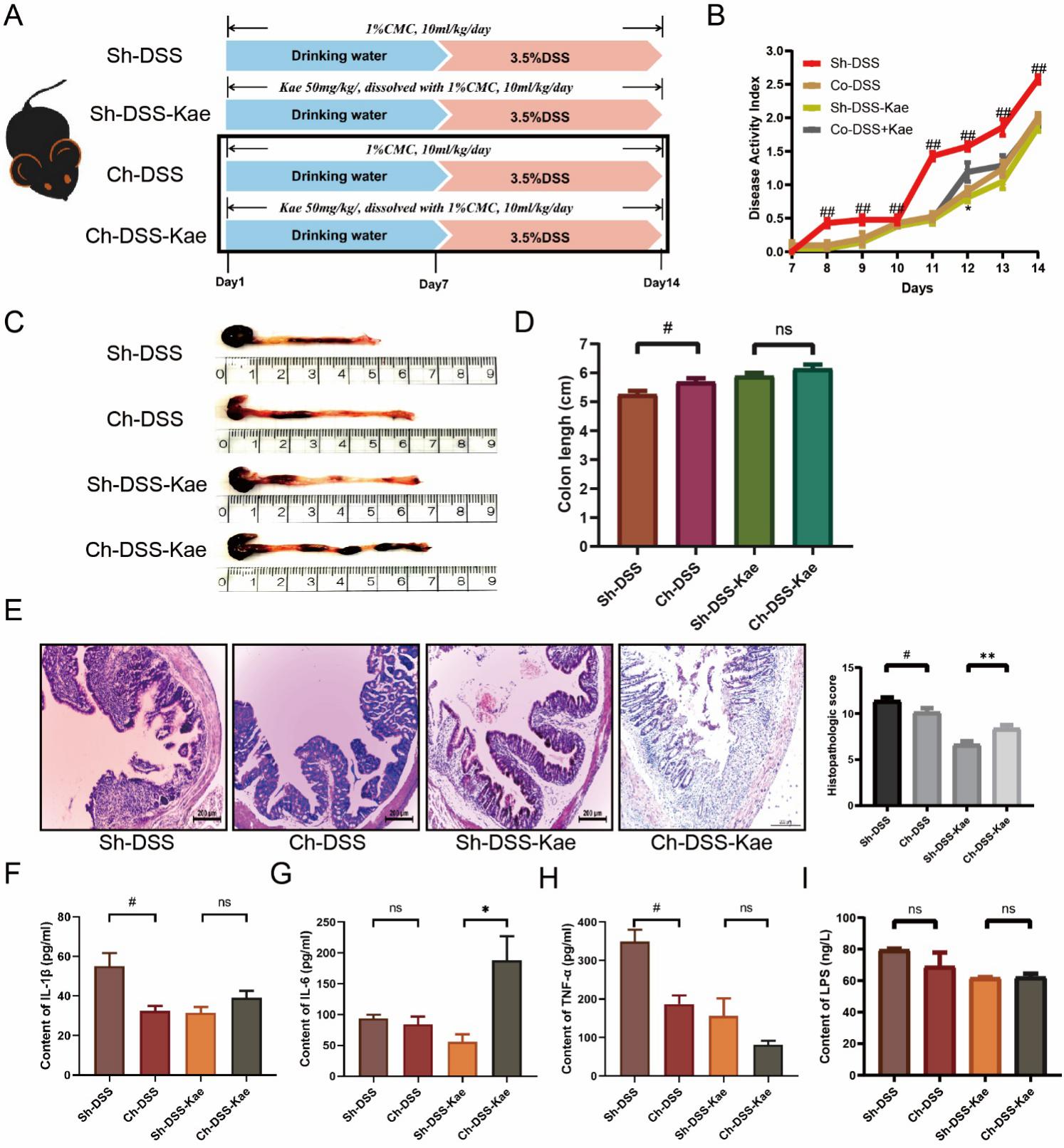
**

**Supplementary Figure 2.** The symptoms of DSS-induced colitis in mice were alleviated by co-housing with Kae-treated mice. (A) Scheme of the experimental procedure. (B) Disease activity index values of mice during the course of colitis (n = 7/group). (C, D) Representative images of mouse colons at sacrifice and statistical analysis of colon length in each group. (E) Representative images of HE stained colon samples (scale bar, 200 μm) and histological scores of colon tissue samples. (F) IL-1β, (G) IL-6, (H) TNF-α, and (I) LPS content. Data are expressed as the mean ± SEM, n = 4, analyzed using one-way ANOVA with Tukey post-hoc analysis. Ch-DSS (vs. Sh-DSS, ^#^*P* < 0.05, ^##^*P* < 0.01); Co-DSS-Kae (vs. Sh-DSS-Kae, **P* < 0.05, ***P* < 0.01); ns, no significant difference.

# Body weight changes in mice during treatment.

The weight changes of the ten groups of mice are shown in **Supplementary Figure S3**. Mice in the DSS group showed significant weight loss at the study endpoint, whereas a slight reduction in body weight was observed in the DSS-Kae group. The body weights of mice in the F-DSS group were significantly lower than those in the F-DSS-Kae group. In addition, the body weights of mice in the Sh-DSS-Kae and Ch-DSS groups were significantly higher than those in the Sh-DSS and Ch-DSS-Kae groups.


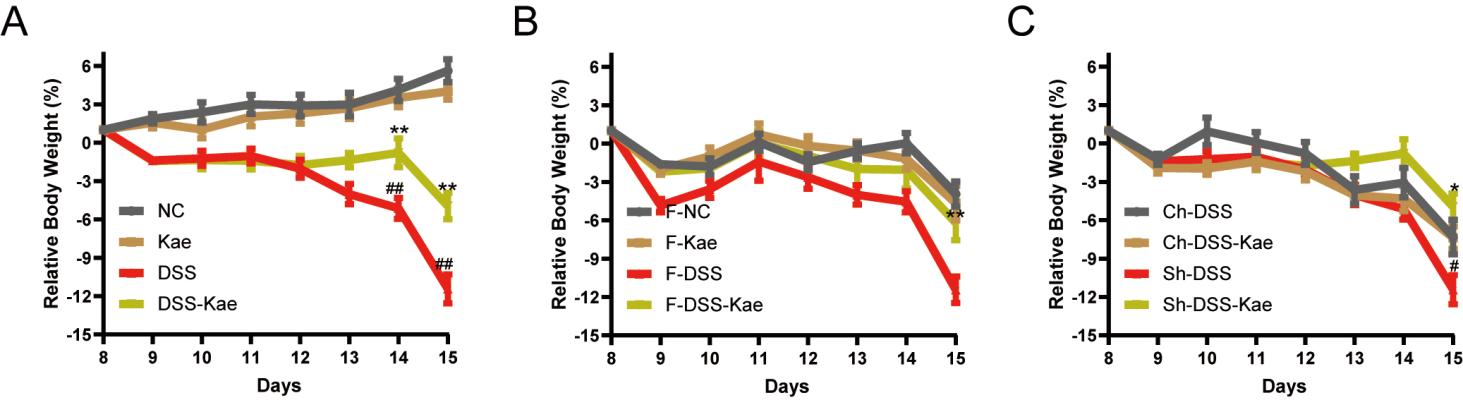


**Supplementary Figure S3.** Changes in body weight of mice in each group during modeling. (A) NC, Kae, DSS and DSS-Kae groups. (B) F-NC, F-Kae, F-DSS and F-DSS-Kae groups. (C) Ch-DSS, Ch-DSS-Kae, Sh-DSS and Sh-DSS-Kae groups. Data are expressed as the mean ± SEM, n = 10, analyzed using one-way ANOVA with Tukey post-hoc analysis. DSS (vs. NC, ^##^*P* < 0.01; vs. DSS-Kae, ***P* < 0.01). F-DSS-Kae (vs. F-DSS, ***P*< 0.01); Ch-DSS (vs. Sh-DSS, ^#^*P* < 0.05,); Co-DSS-Kae (vs. Sh-DSS-Kae, **P* < 0.05).

# Supplementary Tables

**Supplementary Table S1** Composition of the mouse diet

| **Ingredient** | **Unit** | **Content** |
| --- | --- | --- |
| Moisture content | g/kg | 97.0 |
| Crude protein | g/kg | 183.8 |
| Crude fat | g/kg | 56.0 |
| Crude fiber | g/kg | 46.0 |
| Crude ash | g/kg | 73.0 |
| Calcium | g/kg | 12.8 |
| Iron | mg/kg | 160.0 |
| Zinc | mg/kg | 59.0 |
| Carbohydrate | g/100g | 57.0 |
| Energy | kJ/100g | 1563 |

**Supplementary Table S2** Disease activity index (DAI) scores

| **Score** | **Weight loss (%)** | **Stool consistency** | **Occult/gross bleeding** |
| --- | --- | --- | --- |
| 0 | None | Normal | Normal |
| 1 | 1–5 | Loose stools | + |
| 2 | 5–10 | Diarrhea | ++ |
| 3 | 10–15 | Watery stool | Gross bleeding |
| 4 | > 15 | Severe watery stool | Severe/gross bleeding |

Note: + and ++ indicate increasing levels of severity.

DAI = (Weight loss score + Stool consistency score + Occult/gross bleeding score)/3

**Supplementary Table S3** Histopathologic scores

| **Score** | **Inflammatory cells infiltration** | **Depth of invasion** | **Crypt injury** | **Pathological range (%)** |
| --- | --- | --- | --- | --- |
| 0 | None | None | None | 0 |
| 1 | ± | Mucosa layer | 1/3 | 1–25 |
| 2 | + | Mucosa and submucosa | 2/3 | 26–50 |
| 3 | ++ | Full | Full | 51–75 |
| 4 | +++ |  |  | 76–100 |

Note: ±, +, ++, and +++ indicate increasing severity.

**Supplementary Table S4** Primer sequences used for real-time PCR analysis of gene expression

| **Gene** | **Primer** | **Oligonucleotide sequence (5′-3′)** |
| --- | --- | --- |
| *IL-1β* | Forward | CTTTCCCGTGGACCTTCCAG |
|  | Reverse | AATGGGAACGTCACACACCA |
| *IL-6* | Forward | TGCCTTCTTGGGACTGATGC |
|  | Reverse | TGAAGTCTCCTCTCCGGACT |
| *IL-10* | Forward | CTTACTGACTGGCATGAGGATCA |
|  | Reverse | GCAGCTCTAGGAGCATGTGG |
| *TNF-α* | Forward | CTCATGCACCACCATCAAGG |
|  | Reverse | ACCTGACCACTCTCCCTTTG |
| *TLR4* | Forward | GTGTAGCCATTGCTGCCAAC |
|  | Reverse | TCAGGTCCAAGTTGCCGTTT |
| *NF-κB* | Forward | ATGGCAGACGATGATCCCTAC |
|  | Reverse | CGGAATCGAAATCCCCTCTGTT |
| *NLRP3* | Forward | TCTGCACCCGGACTGTAAAC |
|  | Reverse | CATTGTTGCCCAGGTTCAGC |
| *MAPK1* | Forward | GGTTGTTCCCAAATGCTGACT |
|  | Reverse | CAACTTCAATCCTCTTGTGAGGG |
| *COX-2* | Forward | CATCCCCTTCCTGCGAAGTT |
|  | Reverse | CATGGGAGTTGGGCAGTCAT |
| *MCP-1* | Forward | GCAGGTCCCTGTCATGCTTC |
|  | Reverse | GTGGGGCGTTAACTGCATCT |
| *iNOS* | Forward | CTCGGAACTGTAGCACAGCA |
|  | Reverse | GTTGCCATTGTTGGTGGCAT |
| *ZO-1* | Forward | TCTTGCTGGCCCTAAACCTG |
|  | Reverse | GTTGGGCTGGCTCTGAGAAT |
| *Occludin* | Forward | CACCCCCATCTGACTATGCG |
|  | Reverse | CTGGGTATGATCGCTTGCCA |
| *Claudin-1* | Forward | AGCTGCCTGTTCCATGTACT |
|  | Reverse | CTCCCATTTGTCTGCTGCTC |
| *GAPDH* | Forward | AGGTCGGTGTGAACGGATTTG |
|  | Reverse | GGGGTCGTTGATGGCAACA |
